# Strukturen, Ziele und Bedürfnisse der Early Career Scientists am Deutschen Zentrum für Psychische Gesundheit

**DOI:** 10.1007/s00115-024-01631-7

**Published:** 2024-04-26

**Authors:** Shuyan Liu, Simone C. Behrens, Bianca Besteher, Edda Bilek, Woo Ri Chae, Vera Clemens, Christoph Korn, Verena Pflug, Anni Richter, Janine Selle, Julie L. O’Sullivan, Ruth von Brachel, Christina Totzeck, Isabel Brandhorst

**Affiliations:** 1 Klinik für Psychiatrie und Psychotherapie (Campus Charité Mitte), Charité – Universitätsmedizin Berlin, Berlin, Deutschland; 2 Deutsches Zentrum für Psychische Gesundheit (DZPG), https://www.dzpg.org/; 3 Psychosomatische Medizin und Psychotherapie, Medizinische Universitätsklinik, Tübingen, Deutschland; 4 Exzellenzzentrum für Essstörungen (KOMET), Tübingen, Deutschland; 5 Klinik für Psychiatrie und Psychotherapie, Universitätsklinikum Jena, Jena, Deutschland; 6 Abteilung für Psychiatrie und Psychotherapie, Zentralinstitut für Seelische Gesundheit, Medizinische Fakultät Mannheim, Universität Heidelberg, Mannheim, Deutschland; 7 Klinik für Psychiatrie und Psychotherapie (Campus Benjamin Franklin), Charité – Universitätsmedizin Berlin, Berlin, Deutschland; 8 Berlin Institute of Health at Charité – Universitätsmedizin Berlin, BIH Biomedical Innovation Academy, BIH Charité Clinician Scientist Program, Berlin, Deutschland; 9 Abteilung für Kinder- und Jugendpsychiatrie/Psychotherapie, Universität Ulm, Ulm, Deutschland; 10 Sektion Soziale Neurowissenschaften, Abteilung für Allgemeine Psychiatrie, Universität Heidelberg, Heidelberg, Deutschland; 11 Forschungs- und Behandlungszentrum für psychische Gesundheit, Ruhr-Universität Bochum, Bochum, Deutschland; 12 Leibniz-Institut für Neurobiologie, Magdeburg, Deutschland; 13 Center for Intervention and Research on Adaptive and Maladaptive Brain Circuits Underlying Mental Health (C-I-R-C), Halle-Jena-Magdeburg, Deutschland; 14 Institut für Psychologie, Martin-Luther-Universität Halle, Halle, Deutschland; 15 Institut für Medizinische Soziologie und Rehabilitationswissenschaft, Charité – Universitätsmedizin Berlin, Berlin, Deutschland; 16 Abteilung für Psychiatrie, Psychosomatik und Psychotherapie des Kindes- und Jugendalters, Universitätsklinikum Tübingen, Osianderstraße 14–16, 72076 Tübingen, Deutschland

**Keywords:** Training, Mentoring, Vernetzung, Mitbestimmung, Bottom-up-Ansatz, Training, Mentoring, Networking, Co-determination, Bottom-up approach

## Abstract

**Hintergrund:**

Early Career Scientists (ECS) sind Akteur:innen des Wandels und treibende Kräfte in der Förderung psychischer Gesundheit. Das Deutsche Zentrum für Psychische Gesundheit (DZPG) ist eine wichtige Initiative zur Begleitung und Unterstützung von Karrieren im Bereich der psychischen Gesundheit.

**Ziel:**

ECS sollen mithilfe des DZPG in einer interdisziplinären und interinstitutionellen wissenschaftlichen Gemeinschaft gefördert und engagiert werden.

**Strukturen, Themen und Initiativen:**

Das ECS-Board, bestehend aus 18 gewählten ECS-Vertreter:innen, spielt hierfür im DZPG eine zentrale Rolle. Die ECS-Kultur gibt den Mitgliedern eine Mitbestimmungsstruktur, unterstützt Ideen und bekennt sich zu Autonomie. Die sog. DZPG-Akademie wurde entwickelt, um Kommunikation und Vernetzung zu erleichtern und Zusammenarbeit zu fördern. Das DZPG vertritt Schlüsselthemen wie Gleichstellung, Vielfalt, Inklusion, Familienfreundlichkeit und Work-Life-Balance. Das DZPG bietet den ECS Möglichkeiten, zeitgemäße und notwendige Fähigkeiten und Kompetenzen zu entwickeln. Es erweitert die bundesweite Unterstützung für ECS um Fördermöglichkeiten, Unterstützung psychischer Gesundheit am Arbeitsplatz sowie Karriereberatung. Das ECS-Board engagiert sich für Patient:innen- und Öffentlichkeitsbeteiligung und -mitwirkung, Wissenschaftskommunikation sowie den Wissenstransfer in verschiedene Bereiche.

**Schlussfolgerungen:**

Das DZPG wird unter Beteiligung der ECS Trainingsprogramme, den Studierenden- und Akademiker:innenaustausch, Forschungskooperationen und die Bündelung von Ressourcen zur Einwerbung von Fördermitteln und Stipendien fördern. Es wird außerdem die Einrichtung von Knotenpunkten für ECS-Netzwerke unterstützen und den Ausbau der internationalen Kompetenz der ECS in Deutschland fördern.

## Abstract (en)

**Background:**

Early career scientists (ECS) are agents of change and driving forces in the promotion of mental health. The German Center for Mental Health (DZPG) is a powerful initiative to guide and support careers in the field of mental health.

**Objective:**

The DZPG aims to make investments to educate, engage, excite, and empower ECS in an interdisciplinary and interinstitutional scientific community.

**Structures, topics and initiatives:**

To achieve this, the ECS Board at the DZPG plays a central role and consists of 18 elected ECS representatives. The ECS culture gives members the right of voice and embraces bottom-to-top ideas and acknowledges autonomy and co-determination. The DZPG academy was developed to facilitate communication and networking and encourage collaboration among ECS members. The DZPG also navigates several key issues, such as equality, diversity, inclusion, family friendliness and work-life balance, which are essential for a functioning research landscape. The DZPG also extends opportunities to ECS to develop skills and competencies that are essential for contemporary ECS. It complements nationwide support for ECS with funding opportunities, mental health support at work, careers advice and guidance activities. Importantly, the ECS Board is committed to patient and public involvement and engagement, scientific communication and knowledge transfer to multiple settings.

**Conclusion:**

The DZPG will contribute to fostering ECS training programs for student and academic exchanges, collaborative research, and pooling of resources to acquire grants and scholarships. It will also support the establishment of hubs for ECS networks and promote the expansion of international competence of ECS in Germany.

## Struktur und Ziele der Early Career Scientists

Das Deutsche Zentrum für Psychische Gesundheit (DZPG) nahm am 01.05.2023 seine Arbeit auf. Es gehört zu den Deutschen Zentren der Gesundheitsforschung (DZG, deutschezentren.de), die der Bündelung der jeweiligen Spitzenforschung dienen und langfristig durch Bund und Länder gefördert werden sollen [5].

**Figure 1:**
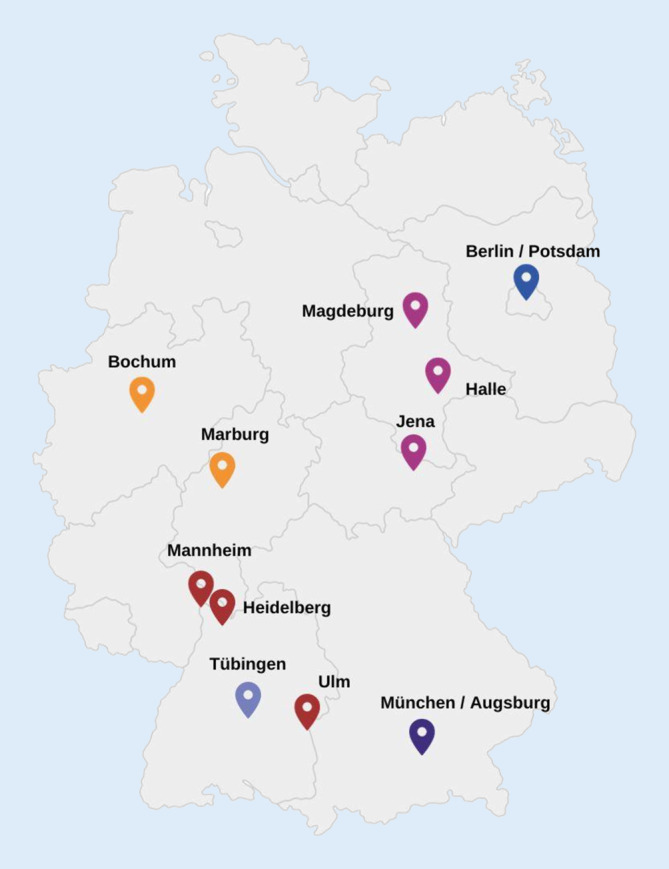

Eine Kernaufgabe des DZPG besteht darin, die nächste Generation von Wissenschaftler:innen und klinisch Tätigen zu fördern, damit diese zu Führungskräften im Bereich der psychischen Gesundheit heranwachsen [8] und so den langfristigen Erfolg des DZPG gewährleisten. Wissenschaftler:innen, die in einer frühen Phase ihrer Karriere stehen, sind im DZPG als Early Career Scientists (ECS) organisiert. Der wichtigste Vermittler zwischen den Wissenschaftler:innen in frühen Karrierephasen und den zentralen Gremien des DZPG ist das ECS-Board. Das Board besteht aus insgesamt 18 gewählten ECS-Vertreter:innen, jeweils 3 ECS-Vertreter:innen der 6 DZPG-Standorte (Abb. 1). Es vertritt die Perspektive der Wissenschaftler:innen in frühen Karrierephasen in Forschungsprojekten und -infrastrukturen und ergreift Initiative für mehr Innovation in zukünftigen Projekten.

**Figure 2:**
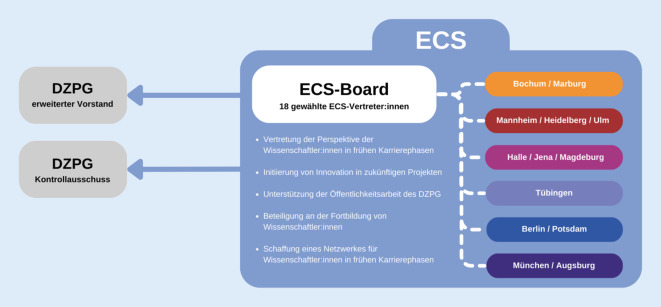

Das ECS-Board delegiert 6 Vertreter:innen in den erweiterten Vorstand (mit Stimmrecht) und in den Kontrollausschuss (mit beratender Funktion) des DZPG, sodass der Aufbau und die Umsetzung des DZPG aktiv von Wissenschaftler:innen in frühen Karrierephasen mitgestaltet werden kann (Abb. 2). Darüber hinaus trägt das ECS-Board zur Öffentlichkeitsarbeit des DZPG bei und wird aktiv an der Fortbildung der Wissenschaftler:innen im Rahmen der Akademie beteiligt sein, u. a. durch den Aufbau eines geplanten Mentoringsystems. Ziel des ECS-Boards ist es auch, ein unterstützendes Netzwerk von und für Wissenschaftler:innen des DZPG in frühen Karrierephasen zu etablieren. So bietet die Organisation allen ECS-Mitgliedern zahlreiche Möglichkeiten an, sich zu vernetzen oder wissenschaftliche Fertigkeiten zu fördern, z. B. in Form gemeinsamer Forschungsprojekte, bei der Mitwirkung von Kongressen oder der Teilnahme an Summer Schools.

Wissenschaftler:innen, die an einem DZPG-Standort oder einer DZPG-Mitgliedseinrichtung angestellt sind, haben die Möglichkeit, sich, abhängig von der jeweiligen Karrierestufe, für zwei Formen der ECS-Mitgliedschaft zu bewerben: als ordentliches oder assoziiertes Mitglied (Tab. 1).

**Table 1:** 

	Assoziiertes Mitglied	Ordentliches Mitglied
Karrierestufe	Promotionsphase	Post-Doc-Phase bis einschließlich W1-Professur
Zeitraum	Bis Abschluss der Promotion	Bis 8 Jahre nach Promotion Mögliche Erweiterungen: – bis zu 5 Jahre für Weiterbildungen und klinische Tätigkeiten – 2 Jahre pro Kind – 2 Jahre für Übernahme einer Pflegetätigkeit, eigene Erkrankung etc.
Angebote	Teilnahme an Kongressen, Netzwerk-Events, Summer Schools etc.	Teilnahme an Kongressen, Netzwerk-Events, Summer Schools etc., zusätzlich wählbar als Standortvertretung und antragsberechtigt für DZPG-Fördermittel

*DZPG *Deutsches Zentrum für Psychische Gesundheit, *ECS* Early Career Scientists

Die Kriterien für eine ECS-Mitgliedschaft wurden in einem kollaborativen Prozess vom ECS-Board erarbeitet und konsentiert (Tab. 1). Diese sind angelehnt an die Kriterien für einen Starting Grant des Europäischen Forschungsrats, wurden jedoch dahingehend angepasst, dass Besonderheiten der ECS im DZPG mit unterschiedlichem fachlichen Hintergrund (z. B. Medizin, Psychologie, Biologie, Ernährungswissenschaft, Medizininformatik) berücksichtigt werden. Insbesondere war es den ECS-Mitgliedern wichtig, dass sehr gut ausgebildete Fachkräfte im Bereich der Psychologie und Medizin, die sowohl wissenschaftlich als auch klinisch tätig sind, im Sinne eines Scientist-Practitioner/Clinician Scientist, keine Nachteile aufgrund ihrer längeren Ausbildungszeit erfahren sollen. Aus diesem Grund wurde das sonst übliche Zeitkriterium für eine Mitgliedschaft von bis zu 8 Jahren nach der Promotion um bis zu zusätzlichen 5 Jahren erweitert, für Weiterbildungen (z. B. psychotherapeutische Ausbildungen/Facharztausbildung) und klinische Tätigkeiten. Den ECS-Mitgliedern ist es zudem ein Anliegen, Wissenschaftler:innen mit Kindern, pflegerischen Verpflichtungen und/oder eigenen Erkrankungen zu unterstützen, sodass pro Kind sowie entsprechend der individuellen zusätzlichen Belastung noch einmal zusätzlich 2 Jahre angerechnet werden können. Ziel ist, die Vielfalt innerhalb der ECS und späteren Führungskräfte zu fördern und besondere Situationen besser berücksichtigen zu können.

Die Anzahl der ECS-Mitglieder je Standort des DZPG ist nicht begrenzt. Eine Mitgliedschaft ist über den Zeitraum von maximal zwei regulären Förderperioden des DZPG – von jeweils 5 Jahren – möglich. Ordentliche Mitglieder haben die Möglichkeit, sich um DZPG-Fördermittel zu bewerben. Damit aber auch Wissenschaftler:innen in der Doktorand:innenphase an den DZPG-Standorten von den vielfältigen Unterstützungsmöglichkeiten der ECS profitieren können (z. B. Teilnahme an Netzwerktreffen), besteht für sie die Möglichkeit, assoziiertes Mitglied zu werden.

## DZPG-Akademie

Die interne Fortbildung und der Aufbau eines Mentoringsystems sind im DZPG im Rahmen einer eigenen Infrastruktur, der DZPG-Akademie, geplant. ECS stellen hier nicht die einzige, aber dennoch eine zentrale Zielgruppe des Angebots dar. Die DZPG-Akademie soll zunächst in den folgenden drei Bereichen entwickelt werden:

- „Educate“,
- „Share“ und
- „Inform“ [8].

Der Bereich „Educate“ umfasst die Entwicklung einer serverbasierten Plattform für die Bereitstellung forschungsrelevanter Kurse zum Thema „Frühe Erkennung, Intervention und Prävention“ und einem Mentoringprogramm mit Modulen zu Karriereentwicklung/Leadership und individueller Betreuung. Der Bereich „Share“ soll über die Entwicklung einer Plattform für strukturierte Vernetzung, etwa einer Angebotsplattform für Lab-Rotations, initiiert werden. Der Bereich „Inform“ soll eine effiziente interne Wissenschaftskommunikation ermöglichen, unter anderem durch ein Podcastformat über Menschen, Projekte und Produkte des DZPG. Die Einbindung der ECS erfolgt strukturell über die Teilnahme von ECS-Vertreter:innen an der inhaltlich koordinierenden Redaktionsgruppe. Zusätzlich soll mit Unterstützung des ECS-Boards insbesondere im Hinblick auf dessen Kommunikations- und Disseminationswege eine breite Beteiligung der ECS bei der Erstellung und Verbreitung erreicht werden.

## Themen und Initiativen der ECS im DZPG

In der aktuellen Aufbauphase des DZPG fokussieren sich die ECS-Mitglieder auf das Ermitteln von Bedarfen und die inhaltliche Konkretisierung der Ziele der Förderung in frühen Karrierephasen. Sie wollen effiziente Prozesse zur Repräsentation und Mitwirkung in den verschiedenen DZPG-Gremien etablieren und die Infrastrukturen durch Gleichberechtigung, Diversität, Inklusion sowie Patient and Public Involvement (PPI) optimieren [2]. Im Allgemeinen stehen die ECS für Folgendes ein:

1. produktive Zusammenarbeit mit Principal Investigators (PIs), PPI und standortübergreifende Synergien,
2. Autonomie und Mitbestimmung,
3. Transparenz bei der Aufgaben- und Ressourcenverteilung an den Standorten.

**Figure 3:**
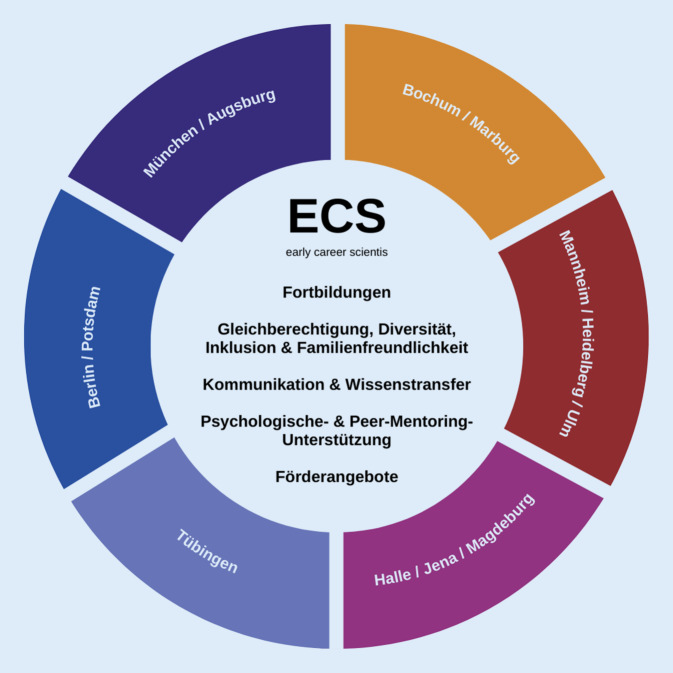

Um weitere Bedarfe zu ermitteln, wird parallel ein Top-down- und Bottom-up-Ansatz verfolgt – d. h., Themen aus den DZPG-Gremien und dem nationalen ECS-Board werden aufgegriffen und bearbeitet, aber auch empirisch ermittelte Bedarfe der ECS erhoben (Abb. 3).

### Befragung von ECS-Mitgliedern: Methodik

Die ECS-Vertreter:innen führten im Herbst 2023 eine anonyme digitale Umfrage an den DZPG-Standorten durch, um die Ziele und Bedarfe der Wissenschaftler:innen in frühen Karrierephasen an den Standorten zu erheben. Dazu wurden Teilnehmende von ECS-Veranstaltungen per QR-Code zur Befragung aufgefordert. Außerdem wurden die ECS-Mitglieder und die assoziierten Mitglieder an allen ECS-Standorten per E‑Mail zur Beantwortung der Befragung gebeten. An der Befragung nahmen 80 Personen teil, 77 vollständige Datensätze gingen in die Ergebnisdarstellung ein. Die Zugehörigkeit zu den ECS (assoziiert oder ordentlich) wurde per Selbstauskunft erhoben (d. h., es wurde nicht überprüft, ob es sich bei den Teilnehmenden der Befragung tatsächlich um eingetragene assoziierte oder ordentliche Mitglieder handelt). Neben persönlichen Angaben und Informationen zum beruflichen Hintergrund (Tab. 2) beantworteten die Teilnehmenden Fragen zu ihren Interessen im Rahmen des DZPG.

Die folgenden Ergebnisse werden für die Gruppe der ordentlichen ECS-Mitglieder (Post-Docs, Habilitierte, Juniorprofessor:innen) und der assoziierten Mitglieder (in der Regel Doktorand:innen) getrennt dargestellt. Dabei werden die von den Autor:innen ausgewählten qualitativen Ergebnisse in den folgenden Abschnitten direkt inhaltlich eingeordnet.

**Table 2:** 

	Assoziierte Mitglieder	Ordentliche Mitglieder
	*n* = 29	*n* = 48
*Standorte:*		
Berlin/Potsdam	8 (27,6 %)	12 (25,0 %)
Bochum/Marburg	6 (20,7 %)	9 (18,7 %)
Heidelberg/Mannheim/Ulm	8 (27,6 %)	8 (16,7 %)
Jena/Halle/Magdeburg	3 (10,3 %)	11 (22,9 %)
München/Augsburg	0 (0,00 %)	2 (4,2 %)
Tübingen	4 (13,8 %)	6 (12,5 %)
*Alter (Jahre±SD):*	30,4 (2,87)	36,3 (4,96)
*Geschlecht:*		
Männlich	7 (24,1 %)	13 (27,1 %)
Nonbinär	1 (3,45 %)	0 (0,00 %)
Keine Angabe	1 (3,45 %)	0 (0,00 %)
Weiblich	20 (69,0 %)	35 (72,9 %)
*Geburtsland:*		
Anderes Land	5 (17,3 %)	6 (12,6 %)
Deutschland	23 (79,3 %)	41 (85,5 %)
Keine Angabe	1 (3,4 %)	1 (2,1 %)
*Elternschaft:*		
Kinder	3 (10,3 %)	24 (50,0 %)
Keine Kinder	26 (89,7 %)	24 (50,0 %)
*Behinderung:*		
Nein	20 (69,0 %)	41 (85,4 %)
Keine Angabe	1 (3,4 %)	1 (2,1 %)
Ja	8 (27,6 %)	6 (12,5 %)
*Abschluss:*		
Diplom/Master	27 (93,1 %)	0 (0,00 %)
Doktor	1 (3,45 %)	40 (83,3 %)
Habilitation	0 (0,00 %)	4 (8,3 %)
Juniorprofessur	0 (0,00 %)	1 (2,1 %)
Staatsexamen	1 (3,45 %)	3 (6,3 %)
*Psychotherapieausbildung:*		
Abgeschlossen	4 (13,9 %)	21 (43,8 %)
Laufend	8 (27,6 %)	9 (18,8 %)
Nein	17 (58,5 %)	18 (37,6 %)
*Angestrebte Professur:*		
Vielleicht	11 (37,9 %)	18 (37,5 %)
Nein	8 (27,6 %)	8 (16,7 %)
Ja	8 (27,6 %)	22 (45,8 %)
Keine Angaben	2 (6,9 %)	–
*Art der Tätigkeit:*		
Nur klinische Tätigkeit	2 (6,9 %)	6 (12,5 %)
Nur nichtklinische Tätigkeit	16 (55,2 %)	30 (62,5 %)
Sowohl klinische als auch nichtklinische Tätigkeit	11 (37,9 %)	11 (22,9 %)
Keine Angabe	–	1 (2,1 %)

*ECS* Early Career Scientists

### Fortbildungen

Die Bedarfe in Bezug auf Fortbildungen waren in beiden Gruppen hoch, unterschieden sich aber thematisch. Insgesamt gaben 77 % der Befragten an, Interesse an einer Summer School zu haben, mit dem Fokus auf die Erweiterung von Fertigkeiten beim Präsentieren, beim Verfassen von Motivationsschreiben, der Erstellung eines Lebenslaufes oder dem Setzen von Prioritäten. Allerdings stießen diese Themen auf mehr Interesse bei den assoziierten Mitgliedern (86 %, *n* = 25) als bei den ordentlichen Mitgliedern (70 %, *n* = 34). Die Interessensgebiete für Fortbildungen der ordentlichen Mitglieder bezogen sich beispielsweise auf die Karriereentwicklung (z. B. Berufungsverfahren), Forschungsmethoden (z. B. Einsatz künstlicher Intelligenz, statistische Methoden) und Mitteleinwerbung.

### Gleichberechtigung, Diversität, Inklusion, Familienfreundlichkeit

Die Implementierung von Maßnahmen zur Förderung von Gleichberechtigung, Diversität, Inklusion und Kinder- und Familienfreundlichkeit ist ein allgemeines und grundsätzlich breit verankertes Ziel des DZPG. Die ECS sind hiervon in besonderem Maße betroffen, was sich nicht nur in der durchgeführten Umfrage, sondern auch in aktuellen Zahlen zur Repräsentanz von Frauen in den verschiedenen Karrierestufen zeigt [4].

Die Teilnehmenden der ECS-Umfrage wurden daher in einem offenen Textfeld zu ihren Ideen befragt, wie das DZPG die psychische Gesundheit der Mitglieder und Familienfreundlichkeit unterstützen könnte. Wiederholt wurden folgende Ideen formuliert:

Ideen zur Förderung der psychischen Gesundheit:

- Arbeitsdruck durch bessere Arbeitsbedingungen reduzieren (z. B. längere Laufzeit von Projekten, längere Verträge, realistischere Arbeitszeiten),
- Austausch zur psychischen Gesundheit fördern (z. B. Supervisions‑/Intervisionsgruppen, Mentoring, Coaching),
- Workshops zu Work-Life-Balance und Burnout,
- offenes Arbeitsklima, in dem über psychische Gesundheit und Probleme gesprochen werden kann (besonders durch Vorgesetzte mit Vorbildfunktion).

Ideen zur Familienfreundlichkeit:

- dienstliche Besprechungen während der vertraglichen Arbeitszeiten ansetzen,
- flexible Arbeitszeiten,
- Unterstützung der Kinderbetreuung (z. B. Übernahme von Reisekosten für Begleitpersonen).

Jedoch ist anzumerken, dass Handlungsfelder zur Verbesserung dieser Situation komplex sind und oft nicht im Verantwortungsbereich des DZPG liegen, etwa bei Themen der Kinderbetreuung und der Ermöglichung finanzieller Förderung von Mitarbeitenden mit Care-Aufgaben oder anderen Zusatzbelastungen. Die besondere Herausforderung liegt daher darin, existierende und entstehende Fördermöglichkeiten vollumfänglich auszuschöpfen und auf die konsequente Implementierung zusätzlicher Maßnahmen zu drängen. Als essenziell betrachten die ECS daher die Etablierung einer insgesamt offenen und flexiblen Arbeitskultur, die besondere Bedürfnisse von Wissenschaftler:innen berücksichtigen kann.

Die Angaben zur aktuellen Tätigkeit der ECS weisen außerdem darauf hin, dass die Erhöhung des Anteils von Scientist-Practitioner/Clinician Scientists unter den ECS ein dringendes Thema ist. Bereits aktuell zeichnet sich ab, dass die wissenschaftliche Tätigkeit insbesondere für Mediziner:innen schwierig umzusetzen ist und sich der Anteil klinisch und wissenschaftlich tätiger Personen in höheren Karrierestufen reduziert. Es ist zu erwarten, dass sich dieses Bild mit der Umsetzung der Reform der Psychotherapieausbildung [1, 7] verschärfen wird, da aktuelle Versionen der Weiterbildungsordnung wenig Raum für gleichzeitige wissenschaftliche Tätigkeiten vorsehen. Die ECS werden daher darauf hinwirken, dass sich das DZPG politisch für angemessene Rahmenbedingungen für Scientist-Practitioner/Clinician Scientists einsetzt.

### Kommunikation und Wissenstransfer

Insgesamt zeigte sich bei allen Teilnehmenden ein großes Interesse an gemeinsamen Forschungsaktivitäten und Netzwerken des DZPG. Beispielsweise berichteten 92 % der Teilnehmenden, an dem Aufbau einer Studie im Rahmen des DZPG interessiert zu sein (ordentliche Mitglieder: 96 %, *n* = 46; assoziierte Mitglieder: 86 %, *n* = 25). 75 % äußerten Interesse oder möglicherweise Interesse an einem Forschungsaufenthalt an einem anderen Standort (ordentliche Mitglieder: 71 %, *n* = 34; assoziierte Mitglieder: 83 %, *n* = 24). 83 % beschrieben ein Interesse an einem Mentor:innenprogramm (ordentliche Mitglieder: 83 %, *n* = 40; assoziierte Mitglieder: 83 %, *n* = 24).

Die externe Kommunikationsstrategie in der psychischen Gesundheitsforschung spielt für die ECS-Mitglieder als Wissenschaftler:innen in frühen Karrierephasen eine entscheidende Rolle bei der Förderung der Translation, der Steigerung der psychischen Gesundheitskompetenz in der Bevölkerung, der Verbreitung von Informationen und der Vermittlung evidenzbasierter Forschungsergebnisse. Insbesondere im digitalen Raum existieren bereits zahlreiche Informationen zu psychischen Erkrankungen, jedoch weist die Qualität dieser eine hohe Varianz auf. Durch den Zusammenschluss engagierter Wissenschaftler:innen in frühen Karrierephasen im Rahmen des DZPG entsteht so die Möglichkeit, hochwertige Informationen seriöser Quellen bereitzustellen.

Um diese Ziele zu erreichen, ist aus Sicht der ECS eine multidimensionale Herangehensweise erforderlich. Zunächst werden die ECS verstärkt auf eine klare und verständliche Kommunikationsform setzen, um komplexe wissenschaftliche Erkenntnisse einem breiteren Publikum zugänglich zu machen. Dies kann durch die Nutzung leicht verständlicher Sprache und visueller Darstellungen gefördert werden. Beispielsweise wären Kurzzusammenfassungen neuer Forschungserkenntnisse in laienverständlicher Sprache oder in visueller Darstellung, wie sie international bereits häufiger verwendet werden, eine denkbare Herangehensweise. Darüber hinaus ist eine gezielte Nutzung digitaler Medien und sozialer Plattformen unerlässlich, um eine größere Reichweite zu erzielen und evidenzbasierte Forschungsergebnisse effektiv zu vermitteln. Diese Strategie werden die ECS auf aktuelle wissenschaftliche Erkenntnisse und die Bedürfnisse der Zielgruppen ausrichten, um eine nachhaltige Verbesserung des Wissens über psychische Gesundheitsfaktoren zu ermöglichen. Damit wird auch das Ziel verfolgt, zur Entstigmatisierung psychischer Störungen beizutragen.

Hinsichtlich der internen Kommunikationsstrategie fördern die ECS eine offene und transparente Kommunikationskultur, in der Ideen und Anliegen der Wissenschaftler:innen in frühen Karrierephasen frei ausgetauscht werden können. Die regelmäßigen Treffen und interdisziplinäre Zusammenarbeit erleichtern den Informationsfluss und eröffnen damit neue Möglichkeiten, Forschungsprojekte gezielt voranzutreiben. Die Einbindung von Doktorand:innen vergrößert die Vernetzungsmöglichkeiten und den direkten Austausch von Erfahrungen in den diversen Qualifikationsstufen.

Darüber hinaus werden die oben beschriebenen Ressourcen und Unterstützungen für Wissenschaftler:innen in frühen Karrierephasen bereitgestellt, um die Anerkennung und Wertschätzung der Arbeit junger Forscher:innen zu betonen, Motivation und Engagement zu fördern und somit die interne Kommunikation in diesem wichtigen Forschungsfeld zu stärken.

### Psychologische- und Peer-Mentoring-Unterstützung

Aktuelle Erhebungen legen nahe, dass etwa ein Drittel der Doktorand:innen unter klinisch signifikanten Symptomen von Angst und Depression leiden [3]; eine Situation, die sich während der SARS-CoV-2(„severe acute respiratory syndrome coronavirus type 2“)-Pandemie noch verschlechtert hat [6]. Auch 20 % der Post-Docs berichteten in einer Erhebung der Max Planck Gesellschaft 2022 von mittelschweren bis schweren depressiven Symptomen (https://www.forschung-und-lehre.de/karriere/postdoc/zahlreiche-postdocs-berichten-von-depressiven-symptomen-5681).

Diese Zahlen sind zwar nicht detailliert auf die Situation des DZPG übertragbar, weisen aber doch darauf hin, dass hier ein deutlicher Bedarf an Erhebungen, präventiven und kurativen Konzepten besteht. Die ECS wollen erreichen, dass sich das DZPG als Arbeitgeber in diesem Bereich besonders engagiert, und entsprechende Initiativen breit in den Strukturen verankern, um als Vorbild für andere Forschungsverbünde zu fungieren und die psychische Gesundheit der eigenen Mitarbeitenden zu schützen und zu fördern.

### Förderangebote

Die ECS können am jeweiligen Standort Mittel über ein eigenes Budget beantragen. Möglich ist beispielsweise die Kostenübernahme von Sach- oder Reisemitteln für Projektpilotierungen und -vorstellungen auf Konferenzen, Laborbesuchen oder Publikationen. Antragsteller:innen müssen den Bezug ihrer Projekte zu den Zielen des DZPG darstellen sowie den inkrementellen Nutzen der Förderung für ihre persönliche Karriereentwicklung darlegen. Der Austausch zwischen verschiedenen Standorten wird besonders positiv bewertet. Über die Anträge entscheidet ein lokales ECS-Board aus Vertreter:innen der am Standort beteiligten Institutionen. Es werden zusätzlich Konzepte erarbeitet, wie ECS sich mit eigenen Forschungsideen in größeren laufenden Projekten einbringen können.

Die ECS sind Akteur:innen des Wandels, zukünftige Führungskräfte und treibende Kräfte bei der Förderung der psychischen Gesundheit im Rahmen des DZPG. Daher werden sich Investitionen in ihre Bildung, ihr Engagement und ihre Begeisterung jetzt und in Zukunft lohnen.

## Fazit für die Praxis

- Laut der repräsentativen Umfrage im Herbst 2023 handelt es sich bei den Early Career Scientists (ECS) des Deutschen Zentrums für Psychische Gesundheit (DZPG) um eine Gruppe von hoher Diversität mit unterschiedlichen Karrierewegen und Ausbildungsbedarfen, jedoch gemeinsamen Werten, die eine Modernisierung der wissenschaftlichen Arbeitskultur erfordern.
- *Fortbildung:* Es besteht ein sehr hohes Interesse (77 %), je nach Ausbildungsstand Fertigkeiten wissenschaftlicher Leitungspositionen zu vertiefen. Summer Schools und engmaschige Symposien sollten dazu angeboten werden.
- *Psychische Gesundheit:* Zur Förderung einer gesunden und nachhaltigen Arbeitsweise sind bessere Arbeitsbedingungen der ECS und ihrer Führungskräfte mit Vorbildfunktion erforderlich(z. B. im Hinblick auf Entfristungen, Arbeitszeitmanagement, offene Kommunikation), aber auch Workshops und Intervisionsgruppen zur Information und zum Austausch über psychische Belastungsfaktoren bei der Arbeit.
- *Familienfreundlichkeit*: Es werden flexiblere und einhaltbare Arbeitszeiten gefordert sowie die Erleichterung von Kongressteilnahmen durch Unterstützung der Kinderbetreuung.
- *Vernetzung:* Fast alle Befragten (92 %) haben ein hohes Interesse an einer standortübergreifenden Zusammenarbeit der ECS im Rahmen von Forschungsaufenthalten und regelmäßigen digitalen und Präsenztreffen.
